# Perception, practice and associated factors of labour pain management among obstetric care providers in public health facilities in Harari Region, Ethiopia: a multicentre cross-sectional study

**DOI:** 10.1093/inthealth/ihae084

**Published:** 2024-11-21

**Authors:** Loza Wondimu, Miressa Bekana, Abera Kenay Tura, Tamirat Getachew

**Affiliations:** School of Medicine, College of Health and Medical Sciences, Haramaya University, Harar, Ethiopia; School of Medicine, College of Health and Medical Sciences, Haramaya University, Harar, Ethiopia; School of Nursing and Midwifery, College of Health and Medical Sciences, Haramaya University, Harar, Ethiopia; School of Nursing and Midwifery, College of Health and Medical Sciences, Haramaya University, Harar, Ethiopia

**Keywords:** eastern Ethiopia, labour pain management, perception, practice

## Abstract

**Background:**

Although providing relief from labour pain can improve the mother's satisfaction with the birthing process and lead to better reproductive outcomes. This study aims to evaluate the perceptions, practices and related aspects of labour pain management among obstetric care providers in public health institutions in the Harari Region of Ethiopia.

**Methods:**

A cross-sectional study was conducted in the labour and delivery wards of public health facilities in the Harari Region. Data were collected using a semi-structured and pretested self-administered questionnaire among systematically selected obstetrics care providers (OCPs). A multivariate logistic regression model was used to identify factors associated with the practice of labour pain management. Odds ratios (ORs) with 95% confidence intervals (CIs) were used to determine the strength of associations and a p-value <0.05 was considered significantly associated.

**Results:**

Data from 234 OCPs were employed for analysis. A total of 70.9% of OCPs have positive perceptions and 69.7% practiced labour pain management in the past 4 weeks. Being a female (adjusted OR [aOR] 2.33 [95% CI 1.06 to 5.15]), having a positive perception (aOR 7.76 [95% CI 3.21 to 18.72]), being a physician (aOR 6.35 [95% CI 1.94 to 20.82]), being a midwife (aOR 5.28 [95% CI 1.78 to 15.63]) and being a highly qualified OCP (aOR 17.89 [95% CI 5.22 to 61.30]) were associated with being more likely to practice labour pain management. Positive attitude (aOR 2.77 [95% CI 1.51 to 8.72]), being a physician (aOR 6.01 [95% CI 1.23 to 29.35]) and practicing labour pain management (aOR 12.89 [95% CI 4.57 to 36.38]) were associated with labour pain management perceptions.

**Conclusions:**

Seven of ten OCPs practiced labour pain relief methods and had a positive perception of managing labour pain. Therefore, facilitating training and improving the perceptions and attitudes of OCPs towards labour pain management should be encouraged.

## Introduction

The pain experienced during childbirth is often considered to be the most excruciating pain that a person can endure.^1^ Most nulliparous women described labour pain as intolerably bad.^2^ An individual's emotional, motivational, cognitive, social and cultural circumstances can all impact the pain experienced during labour.^3^ An emphasis is placed on a woman-centred approach to managing labour pain due to awareness of labour pain in a multidimensional framework.^4^

A wide range of pharmacological and non-pharmacological techniques are used for managing labour pain.^4^ While pharmacological interventions focus on addressing the current pain, non-pharmacological interventions generally attempt to assist women in coping with labour pain.^5^ Labor pain can cause physical agony, physiological changes, emotional misery and despair for women during and after delivery. These issues can affect the mother and the unborn child.^4^

There is no other situation in which it is deemed appropriate for a person to feel extreme pain, amenable to safe pain medication, while under a doctor's care, according to the American College of Obstetricians and Gynaecologists (ACOG). The idea that a mother's request alone constitutes a valid medical justification for labour analgesia is supported by the ACOG and the American College of Nurse-Midwives.^6^

In high-income nations, pain management solutions are more widely available and have become an essential component of the labour process. In low-income areas, the situation is different and labour pain treatment is frequently disregarded. The identified barriers to providing labour pain management techniques, particularly pharmaceutical ones, include lack of understanding among women and obstetrics care providers (OCPs), rejection, lack of availability and safety concerns.^7^ Studies in Nigeria indicate that most OCPs have a positive perception of labour pain management.^7,8^ However, in Ethiopia, only 57.2%^9^ and 24.9%^10^ of OCPs working in Amhara and Tigray regional states, respectively, perceived that pain during labour should be managed.

In Africa, the practice of labour pain management is often neglected. A study performed by Ogboli et al.^8^ found that only 48.4% of OCPs had administered pain relief during labour in the preceding 4 weeks. Similarly, a study of all OCPs in Amhara Regional State referral hospitals revealed that just 40.1% of them used obstetric analgesia regularly.^11^

Negative perceptions of OCPs towards labour pain management have resulted in poor utilization of labour pain management choices and generally inadequate intrapartum pain management. Despite the national commitment to promote pain-free labour and the recognition of pain relief as an essential component of intrapartum care, there is a scarcity of studies on clinicians’ attitudes and practices toward labour pain management.^11^ Although the use of labour pain management techniques and their integration into the care delivery system is not a priority in underdeveloped countries like Ethiopia, alleviating the pain of the labouring mother can significantly impact the consumption of obstetric care services. Thus the study aimed to assess perceptions, practices and associated factors of labour pain management among OCPs working at labour and delivery units in public health facilities in Harari Region, Ethiopia.

## Methods

### Study area and period

From 2 to 30 November 2020, a survey was conducted in public health facilities offering healthcare services in Harari Regional State, in eastern Ethiopia. The distance between Addis Ababa, the Ethiopian capital city, and Harar is 525 km and 246 000 people call Harari home. The region's health services include 26 health posts, 21 private clinics, 8 health centres (HCs), 7 hospitals and 1 regional laboratory.

### Study design and population

A cross-sectional study was conducted among all OCPs working in the study area who had attended at least one delivery in the past month. Health professionals who were present in the labour and delivery units solely for consultation (senior staff who only visited clients/patients for consultation without being employed at the facility) were not included in the study.

### Sample size determination and sampling procedure

All identified OCPs (n=250) working at the labour and delivery units in the study area during the study period were included. The study was conducted in 10 public health facilities: Hiwot Fana Specialized University Hospital, Jugel Hospital, Aboker Health Center, Jine'ala Health Center, Amir-Nur Health Center, Hakim Health Center, Harewe Health Center, Hasen-Gey Health Center, Sigicha Health Center and Erer Health Center. Of a total of 562 OCPs working in these public health facilities during the study period, 250 were working in the labour and delivery units and all of them were included.

### Data collection procedure

The data were collected using a self-administered structured questionnaire adapted from previous studies.^10,12^ The questionnaire included sociodemographic characteristics of the OCPs, including age, gender, health facility, working experience and training in pain relief methods, participants’ perception and attitude towards labour pain relief methods, participants’ use of labour pain relief methods in the past 4 weeks and barriers to using labour pain relief methods in healthcare settings, using a 5-point Likert scale (strongly agree, agree, neutral, disagree and strongly disagree). Ten trained health professionals (BSc; seven midwives and three nurses) collected the data under the supervision of four midwives (MSc).

### Measurement and operational definitions

Perception was measured from a total of 11 items on the Likert scale ranging from 0 to 5. Positive perception was study participants who scored less than the mean value and negative perception was study participants who scored greater than the mean value. Overall practice was the proportion of OCPs who reported managing labour pain (pharmacologic or non-pharmacologic) at least once in the 4 weeks prior to the study.

### Knowledge

A total of 15 items were used to assess knowledge of labour pain management methods. The questions were answered with ‘yes’, ‘no’ or ‘I don't know’ responses. During analysis, the correct answer was coded as 1 and an incorrect or I don't know answer was coded as 0. The sum score (range 0–15) was then computed and categorized. Adequate knowledge was defined as OCPs who answered ≥6.65 (mean value) on the knowledge-related labour pain relief method questions,^13^ inadequate knowledge was defined as OCPs who scored <6.65^13^ and highly qualified health professionals are obstetricians/gynaecologists who are experts in managing labour pain and can administer or recommend various pain relief methods, including epidurals and medications.

### Data quality control

Training was provided for data collectors and supervisors to provide a common understanding of the study in general and the questionnaire in particular. The questionnaire was pre-tested in a Federal Police Hospital in the nearby area.

### Data processing and analysis

The data were coded, cleaned and edited before being entered into EpiData version 3.1 (EpiData Association, Odense, Denmark). Descriptive analysis using SPSS version 25 (IBM, Armonk, NY, USA) stat calc was presented with proportion and 95% confidence interval (CI). Continuous variables were described using mean and standard deviation (SD). A χ^2^ assumption was checked before fitting the model. The Hosmer–Lemeshow statistic and omnibus test were checked to ensure the model's goodness of fit and multicollinearity was diagnosed using the variance inflation factor test. A binary logistic regression model was utilized to detect the association between independent variables and the outcome variable. All variables with a p-value ≤0.25 in the bivariate analysis were exported to multivariate analysis to control all possible confounders. The degree of the statistical association between determinants and outcome variables was assessed by using an odds ratio (OR) at a 95% level of confidence. Statistical significance was set at p<0.05.

### Ethical considerations

Ethical clearance was secured from the Institutional Health Research Ethics Review Committee (IHRERC) of Haramaya University College of Health and Medical Sciences before data collection. In addition, official permissions were received from facility administration and inpatient coordinators. Before the interview, informed voluntary written and signed consent was obtained from each study participant. All World Health Organization–recommended protective measures, such as one-at-time questionnaire distribution, social distancing and wearing face masks by both the data collectors and the study participants, were followed during data collection.

## Results

### Sociodemographic characteristics of respondents

Of the 250 OCPs surveyed, 234 (93.6%) were employed for the analysis. The mean age of the respondents was 28 y (SD 5.4). The majority were males (144 [61.5%]), 20–29 y of age (170 [72.6%]) and had clinical experience of <5 y (177 [75.6%]). Roughly half (124 [53%]) of the respondents had a BSc degree, were midwives (120 [51.3%]) and were ever married (129 [55.1%]) (Table 1).

**Table 1.** Sociodemographic characteristic of OCPs working in labour and delivery units in public health facilities in Harari Region, Ethiopia, 2020 (n=234)

Characteristics	n	%
Age (years)		
20–29	170	72.6
30–39	44	18.8
≥40	20	8.5
Sex		
Male	144	61.5
Female	90	38.5
Ethnicity		
Oromo	135	57.7
Amhara	56	23.9
Harari	20	8.5
Gurage	9	3.8
Tigre	7	3.0
Other	7	3.0
Religion		
Muslim	52.6	52.6
Orthodox	34.6	34.6
Protestant	11.1	11.1
Other	1.7	1.7
Marital status		
Married	123	52.6
Single	105	44.9
Divorced	4	1.7
Widowed	2	0.9
Level of education		
Diploma	25	10.7
BSc	101	43.2
Masters	24	10.3
MD/specialist/resident/medical intern	84	35.8
Profession		
Midwife	121	51.7
Nurse	16	6.8
Health officer	6	2.6
IESO	3	1.3
Physician	84	35.9
Other^a^	4	1.7
Clinical experience (years)		
≤5	177	75.6
6–9	26	11.1
≥10	31	13.3

a Anaesthesiologist, resident, medical doctor.

### Knowledge about labour pain relief methods

Labour pain management methods were known by all OCPs. The most commonly identified method was psychotherapy (reassurance, encouragement and showing compassion), reported by 228 (97.4%). The least known methods were transcutaneous electrical nerve stimulation (47 [20.1%]) and inhalational anaesthesia (32 [13.6%]) (Table 2).

**Table 2.** Knowledge of labour pain management options among OCPs working in labour and delivery units in public health facilities in Harari Region, Ethiopia, 2020 (n=234)

Type	n	%
Psychotherapy	228	97.4
Massage the back	206	88.0
Allowing ambulation	189	80.8
Allowing companions of labouring women choice	163	69.7
Showing how to bear down	151	64.5
Epidural analgesia	137	58.5
Allowing free vertical positioning	108	46.2
Music therapy	92	39.3
Systemic opioids	90	38.5
NSAIDs	82	35.0
Cervical/local anaesthesia	64	27.4
Acupuncture	57	24.4
Hypnosis	50	21.4
Application of TENS	47	20.1
Inhalational anaesthesia	32	13.7

NSAIDs: non-steroidal anti-inflammatory drugs; TENS: transcutaneous electrical nerve stimulation.

### Pain expectations and personal preferences

A total of 143 (61%) respondents rated labour pain as severe, while 68 (29%) rated it as unbearable. The majority (200 [85.4%]) preferred non-pharmacologic methods for labour pain management. A total of 177 (75.6%) of the participants perceived that labour pain should be managed, of which 88 (49.6%) and 61 (34.6%) indicated always and for women with severe pain, respectively.

**Figure 1.**
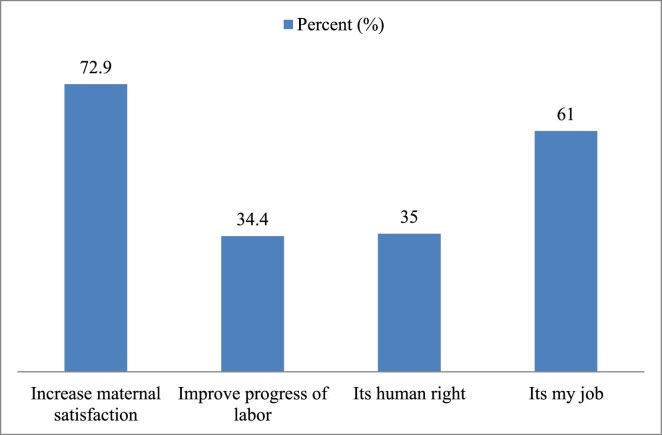
Reasons for offering labour pain management among OCPs in labour and delivery units in public health facilities in Harari Region, Ethiopia, 2020 (n=177).

**Figure 2.**
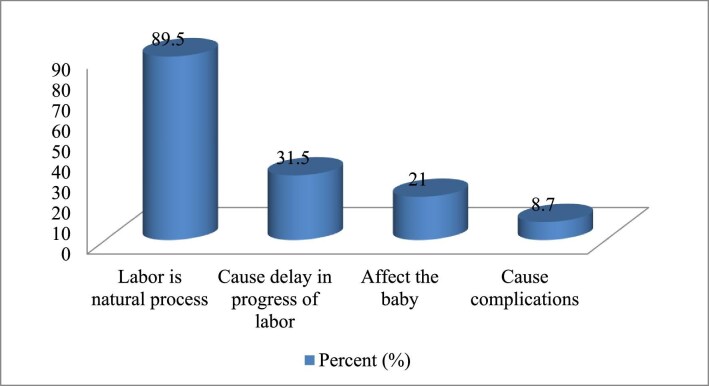
Reasons for not offering labour pain management among OCPs working in labour and delivery units in public health facilities in Harari Region, Ethiopia, 2020 (n=57).

The proponents of labour pain management reported that it increases maternal satisfaction (72.5%). Among those, 77 (24.4%) thought labour pain should not be managed and 69 (89.5%) assumed that labour is a natural process. Generally, 166 (70.9%) had a positive perception towards managing labour pain. More than half of the respondents (129 [55.4%] and 130 [55.8%]) agreed that pharmacological labour pain management affects the foetus and labour progress, respectively. More than two-thirds of the respondents reported that the high flow of patients influences the practice of labour pain management (Figures 1 and 2).

### Practice of labour pain management

A total of 163 (69.7%; 95% CI 63.7 to 75.6) OCPs reported that they had provided some form of labour pain management in the past 4 weeks. Psychotherapy (reassurance, encouragement and showing compassion) is the most commonly practiced (146 [96.9%]) form of labour pain management, while pharmacologic options were offered less often (Table 3).

**Table 3.** Labour pain management options practiced by OCPs working in labour and delivery units in public health facilities in Harari Region, Ethiopia, 2020 (n=163)

Type	n	%
Psychotherapy	158	96.9
Allow the mother to ambulate	136	83.4
Massage the back	131	80.3
Show the woman how to bear down	76	46.6
Allow free vertical positioning	19	11.6
Allowed companion of woman's choice	17	10.4
Systemic opioids	12	7.3
Cervical/local anaesthesia	3	1.8
NSAIDs	2	1.2
Epidural analgesia	1	0.6
Overall practice	163	69.7

NSAIDs: non-steroidal anti-inflammatory drugs.

The most commonly reported reasons among the OCPs who did not practice labour pain management (71 [30.3%]) in the past 4 weeks were lack of training and skill (57 [80.3%]), lack of supply (21 [52.4%]), high patient flow (25 [36%]) and considered management not necessary (4 [6%]).

### Factors associated with the practice of labour pain relief methods

To detect factors associated with labour pain management practice, seven variables from sociodemographic, perception items, current practice and knowledge-related factors having a p-value ≤0.25 on bivariate analysis were fitted into the final model. The multivariable logistic regression analysis revealed that labour pain management practice is significantly associated with the OCP's sex, perceptions, profession and level of qualification.

Female OCPs were 2.33 times more likely to practice labour pain relief methods as compared with males (adjusted OR [aOR] 2.33 [95% CI 1.06 to 5.15]). Compared with lower qualification levels, the odds of labour pain management practice was higher among medium and highly qualified OCPs (aOR 4.35 [95% CI 1.73 to 10.94] and aOR 17.89 [95% CI 5.22 to 61.30], respectively).

OCPs who had a positive perception towards labour pain relief methods were seven times more likely to practice labour pain relief methods compared with their counterparts (aOR 7.76 [95% CI 3.21 to 18.72]). Compared with other professionals, obstetric care physicians and midwives were more likely to practice labour pain relief methods (aOR 6.35 [95% CI 1.94 to 20.82] and aOR 5.28 [95% CI 1.78 to 15.63], respectively). The age of the OCP, marital status and believing labour pain relief methods cause foetal distress were not statistically associated with the practice of labour pain relief methods (Table 4).

**Table 4.** Bivariate and multivariate analysis of factors associated with practicing labour pain management among OCPs in Harari Region, Ethiopia (n=234)

		Practiced labour pain relief methods			
Variables	Category	Yes, n (%)	No, n (%)	Crude OR (95% CI)	aOR (95% CI)
Age (years)	20–29	118 (50.4)	52 (22.2)	0.57 (0.18 to 1.78)	0.50 (0.12 to 2.12)
	30–39	29 (12.4)	15 (6.5)	0.48 (0.14 to 1.71)	0.30 (0.06 to 1.50)
	>40	16 (6.8)	4 (1.7)	1	1
Gender	Female	73 (31.2)	17 (7.3)	2.58 (1.38 to 4.82)	2.33 (1.06 to 5.15)*
	Male	90 (38.3)	54 (23.1)	1	1
Qualification level	High	66 (28.2)	6 (2.6)	23.83 (8.09 to 70.18)**	17.89 (5.22 to 61.30)*
	Medium	85 (36.3)	39 (16.7)	4.72 (2.16 to 10.32)**	4.35 (1.73 to 10.94)**
	Low	12 (5.1)	26 (11.1)	1	1
Perceptions	Negative	95 (40.6)	9 (3.8)	1	1
	Positive	68 (29.1)	62 (26.5)	9.62 (4.48 to 20.69)**	7.76 (3.21 to 18.72)**
Health profession	Physician	66 (28.2)	17 (7.3)	7.06 (2.85 to 17.51)**	6.35 (1.94 to 20.82)**
	Midwife	86 (36.8)	34 (14.5)	4.60 (1.99–10.61)*	5.28 (1.78 to 15.63)**
	Other	11 (4.7)	20 (8.5)	1	1
Marital status	Ever married	90 (38.5)	39 (16.7)	1	1
	Never married	73 (31.2)	32 (13.7)	1.01 (0.58 to 1.77)	0.64 (0.30 to 1.38)
Thought it causes foetal distress	Yes	99 (42.3)	33 (14.1)	1.78 (0.59 to 3.13)	0.89 (0.92 to 5.13)
	No	64 (27.4)	38 (16.2)	1	1

Significant at *p<0.05 and **p<0.001.

### Factors associated with the perception of labour pain management methods

The multivariable logistic regression analysis identified that a positive attitude, professional background and practice were significantly associated with the perception of labour pain management methods. Healthcare providers with a positive attitude towards pain relief were nearly three times more likely to perceive labour pain management methods positively (aOR 2.77 [95% CI 1.51 to 8.72]). Physicians, compared with other health professionals, were six times more likely to have a favourable perception of these methods (aOR 6.01 [95% CI 1.23 to 29.35]). Furthermore, those who demonstrated effective practice in labour pain relief were significantly more likely to have a positive perception of labour pain management methods (aOR 12.89 [95% CI 4.57 to 36.38]) (Table 5).

**Table 5.** Bivariate and multivariate analysis of factors associated with the perception of labour pain management among OCPs in Harari Region, Ethiopia (n=234)

		Perception of pain relief methods			
Variables	Category	Positive, n (%)	Negative, n (%)	Crude OR (95% CI)	aOR (95% CI)
Gender	Female	89 (38.0)	55 (23.5)	1.93 (1.13 to 3.30)*	1.78 (0.90 to 3.49)
	Male	41 (17.5)	49 (20.9)	1	1
Age (years)	20–29	96 (41.0)	74 (31.6)	1.06 (0.42 to 2.70)	0.53 (0.20 to 2.27)
	30–39	23 (9.8)	21 (9.0)	0.90 (0.31 to 2.59)	0.39 (0.10 to 1.56)
	>40	11 (4.7)	9 (3.8)	1	1
Clinical experience (years)	≤5	104 (44.4)	73 (31.2)	1.03 (0.48 to 2.23)	1.08 (0.40 to 2.92)
	6–9	8 (3.4)	18 (7.7)	0.32 (0.11 to 0.96)	0.34 (0.09 to 1.32)
	≥10	18 (7.7)	13 (5.6)	1	1
Attitude	Negative	75 (32.1)	24 (10.3)	1	1
	Positive	55 (23.5)	80 (34.2)	4.55 (2.56 to 8.10)**	2.77 (1.51 to 8.72)**
Health profession	Physician	35 (15.0)	48 (20.5)	0.30 (0.12 to 0.73)*	6.01 (1.23 to 29.35)*
	Midwife	73 (31.2)	47 (20.1)	0.64 (0.27 to 1.50)	0.65 (0.08 to 5.63)
	Other^a^	22 (9.4)	9 (3.8)	1	1
Education level	Low	32 (13.7)	6 (2.6)	1	1
	Medium	70 (29.9)	53 (23.1)	4.04 (3.11 to 20.31)*	2.66 (0.78 to 9.10)
	High	28 (12.0)	44 (18.8)	8.38 (1.13–13.68)*	1.35 (0.65 to 2.81)
Good practice	No	62 (26.5)	9 (3.8)	1	1
	Yes	68 (29.1)	95 (40.6)	9.62 (4.48 to 20.69)**	12.89 (4.57 to 36.38)**

Significant at *p<0.05 and **p<0.001.

a Nurse, public health officer or IESO.

## Discussion

Labor pain can be perceived to be the most severe form of pain experienced in a woman's life, but the provision of pain relief during childbirth is often neglected. In this study, 69.7% (95% CI 63.7 to 75.6) of OCPs reported that they practiced labour pain relief methods and 7 of 10 OCPs reported offering at least one labour pain management service to women in the past 4 weeks. Similarly, 70.9% of OCPs had a positive perception of managing labour pain. This has great implications for maternal delivery service satisfaction and psychological distress and may prevent future institutional delivery.^8,14^

The provision of labour pain management in our study was higher than in previous studies conducted in the Tigray Region (43.3%)^10^ and the Amhara Region (34.4%)^9^ in Ethiopia and in a study conducted in Nigeria (48.4%).^8^ This discrepancy might be related to gradual development of the provider's perception, the sample size difference, the health facility set-ups and the presence of skilled and trained OCPs. To enhance labour pain management practice among OCPs, facilitating training, increasing the number of OCPs and improving the knowledge of OCPs should be provided.^8,13^ However, our findings are still lower than the those from India (92%).^15^ This difference may be the result of institutional labour management protocols, the availability of drugs and supervision.

Female OCPs were more likely to manage labour pain compared with males, which contradicts the findings from Hawassa City.^16^ This may be related to the fact that female providers displayed a connection with labouring women and providing women-centred care, including managing labour pain.^17^ Additionally, female OCPs may encounter such pain themselves and know the severity of labour pain.

Highly qualified OCPs were more likely to practice labour pain relief methods. Evidence showed that OCPs with higher qualifications or education levels have better attitudes about the severity of labour pain and are committed to providing labour pain relief methods.^5,18^ Moreover, OCPs with higher education levels had sufficient knowledge and skill that in turn increased the utilization of both pharmacological and non-pharmacologic labour pain management.^18^ Generally, satisfaction with delivery services depends not only on the level of experienced pain, but also on the appropriate care received during labour, which requires qualified personnel.^17^

OCPs with positive perceptions towards labour pain management were more commonly able to practice labour pain management. This finding is supported by previous findings.^12,16,19^ Positive perceptions towards labour pain management may prove helpful in gaining more knowledge and having more realistic service delivery and more frequent use of techniques of pain relief.^20^ Studies revealed that good perceptions and early preparation are necessary to provide a positive approach for women towards labour and more common use of pain relief techniques.^21^

This study found that the level of labour pain management practice and perception was higher among physicians and midwives compared with other professionals, such as nurses, public health officers and integrated emergency surgical officers (IESOs). The degree of success of the pain relief technique used in the facility depends on the availability of maternity staff. Labour pain relief requires teamwork, including the cooperation of the anaesthetist, midwife, nurse and physician. This implies the need to equip all OCPs working in the obstetrics ward with a basic understanding of the subject and the ability to provide and advise women in labour pain management, as the need for alternative treatments in obstetrics is likely to increase.^20^

The findings of this study should be considered in light of the following limitations. The study population included only skilled OCPs who deliver routine obstetrics care, excluding health management units that might have different experiences and attitudes towards labour pain management—an important factor in addressing barriers to implementing quality care, including pain relief. Additionally, the perspectives of labouring women regarding pain relief were not explored. The study used a small sample size and did not use qualitative data, and future research should aim to include both quantitative and qualitative findings.

## Conclusions

Seven in ten OCPs practiced labour pain relief methods and had a positive perception of managing labour pain. The most commonly practiced method was non-pharmacological labour pain management methods. The sex, perceptions, profession and level of qualification of OCPs were found to be significantly associated with labour pain management practice and a positive attitude, professional background and effective practice were significantly associated with the perception of labour pain management methods. All stakeholders must work together to enhance the level of education and improve the perception of OCPs (especially among midwives, nurses, public health officers and IESOs) towards labour pain management.

## Data Availability

Pertinent data are presented in the article. Additional data can be requested from the corresponding author.
